# Measuring patient satisfaction in an outpatient psychiatric clinic. What factors play a role?

**DOI:** 10.1186/s12991-022-00379-1

**Published:** 2022-01-18

**Authors:** Magdalena Romanowicz, Tyler S. Oesterle, Paul E. Croarkin, Bruce Sutor

**Affiliations:** Department of Psychiatry & Psychology, Mayo Clinic-Rochester, 200 First Street SW, Rochester, MN 55905 USA

**Keywords:** Patient satisfaction, Psychiatry, Resident clinic, Depression, Anxiety, Mentalization

## Abstract

**Introduction:**

Patient satisfaction is defined as the perception that one’s general health care needs are being met. Prior research suggests that positive patient satisfaction with health care facilitates the physician–patient relationship and enhances quality of life.

**Objective:**

The primary purpose of this study was to assess patient satisfaction (as measured by the Patient Satisfaction Questionnaire (PSQ-18)) of patients observed by general psychiatry residents and to examine the effects of depression and anxiety on patient satisfaction. A secondary purpose was to explore the effects of three 1-h mentalization-based skills training sessions on the PSQ-18 scores of psychiatric residents. We hypothesized that depressive and anxiety symptoms would negatively impact patient satisfaction. We hypothesized that patients’ satisfaction scores would improve after mentalization training.

**Methods:**

This was a prospective case–controlled study, enrolling adult patients (*n* = 157) referred for psychiatric assessment in a psychiatric resident outpatient clinic. The primary outcome was patient satisfaction as measured by the PSQ-18. This outcome was compared to anxiety and depression symptoms as measured by the Patient Health Questionnaire (PHQ-9) and Generalized Anxiety Disorder 7-Item scale (GAD-7) questionnaires. Outcome data from the PSQ-18 were compared among residents before and after they completed mentalization training. The data were analyzed with univariate analyses and multiple linear regression.

**Results:**

Overall the patients were satisfied with clinician communication and interpersonal manner (4.21 ± 0.66 and 4.15 ± 0.69, respectively). The patients score on PHQ-9 was inversely related to their scores on time spent (TS) (*p* = 0.01) and accessibility/convenience (AC) (*p* = 0.0009) subscales of the PSQ-18. GAD-7 score was inversely related to patients scores on AC subscale (*p* = 0.01). Brief mentalization training for the providers did not impact patient satisfaction scores.

**Conclusions:**

Our study reveals that depression and anxiety can negatively impact PSQ-18 patient scoring in psychiatric outpatients observed for the first time in a resident clinic. However, this study failed to show that a brief mentalization-based training could improve patient satisfaction scores that were already quite high at baseline.

## Introduction

Patient satisfaction is defined as the extent to which patients perceive their general health care and medical needs are being met [1]. Most health care providers are aware of the great importance of patient satisfaction in facilitating the provider–patient relationship. Different instruments have been used to measure satisfaction [2, 3]. Recently, there has been an increased effort to use patient satisfaction as one of the several measures of the overall quality of health care [1, 2], and with the approval of the Affordable Care Act (ACA) in the United States, patient satisfaction is now tied to reimbursement in some cases [3].

The clinician’s ability to explain, listen, and empathize is thought not only to impact patient satisfaction and experience of care but also to affect functional health outcomes [4, 5]. However, there is growing evidence that other factors outside the doctor–patient relationship influence patient satisfaction [6, 7].

Several studies have looked at patient’s satisfaction in psychiatric settings [8–12]. Kelstrup et al. sent 274 German patients a questionnaire concerning satisfaction with psychiatric treatment 1 month after their discharge from a psychiatric hospital. Patients, who were diagnosed as suffering from affective disorders or from reactive psychoses, were more satisfied than patients with schizophrenia, paranoia, or with transitory adjustment reactions. Patients who had no personality disorder diagnosis and patients with character neurosis were more satisfied than patients with antisocial or borderline personality disorders. Patients on antidepressant medication were much more satisfied than other patients [8]. More recently, Gebhardt et al. evaluated 113 German patients at time of discharge from a psychiatric hospital. They found that patient satisfaction was dependent on symptom severity, global functioning at discharge, pharmacologic disturbances during treatment, and on the diagnostic group [11]. An Indian study of 60 individuals utilized a cross-sectional study design to evaluate patient satisfaction in an outpatient setting utilizing the patient satisfaction questionnaire. They also found that patient’s satisfaction was correlated to illness severity [10].

A review of 6 randomized trials of participants with major depression treated with various antidepressant medication found a correlation between patient satisfaction and patient improvement in depressive symptoms [13]. Other studies have noted patient satisfaction correlated to disease type, age, educational level, level of anxiety, and pain level [12, 14, 15]. Many of these factors are out of a providers control on an initial visit; however, improving a provider’s ability to empathically communicate could be a way to impact patient satisfaction despite comorbidities and treatment outcomes [4, 5, 14].

The concept of mentalizing was introduced as a theoretical model in the 1970s [16]. However, more recently the concept of “theory of mind” has matured through neurobiological studies (mirror neurons theory) and developmental psychology [17]. Mentalization, at a very basic level, is defined as an ability to hold another’s mind within one’s own [16]. It comes from a well-defined concept of “empathy” described in psychoanalytic literature by Greenson et al. [18, 19]. The ability to communicate empathically is an important skill in every doctor–patient interaction, including those devoted primarily to prescribing and monitoring medication. Empathetic communication can increase the patient’s perception that they are being understood and getting their needs met, thus improving the patient’s satisfaction with the encounter [4, 5, 20, 22].

This study sought to examine Patient’s Satisfaction Questionnaire (PSQ-18) measures and their relationship to depression and anxiety symptom severity (as assessed with the PHQ-9 and GAD-7). A secondary aim of the study was to assess the impact of physician mentalization training on PSQ-18 outcomes. We hypothesized that the more depressed and anxious the patient, the lower their average provider satisfaction rating. We also hypothesized that provider empathy training based on teaching basic skills of mentalization could improve empathic communication, thus improving patient satisfaction scores.

## Materials and methods

This was a prospective case–controlled study performed among patients being observed for an “initial” psychiatric evaluation in an outpatient resident clinic at a major psychiatric center located in Minnesota. This study was reviewed by Mayo Clinic internal review board (IRB) and found to have minimal risk (i.e., the probability and magnitude of harm or discomfort anticipated in the research were not greater than that ordinarily encountered in the daily life of the general population or during the performance of routine physical or psychological examinations or tests). This study included male and female patients 18 years or older who provided informed consent. Recruitment was performed using a brochure given to the patient by the desk receptionist that described this study and its objectives. This study excluded patients who were unable to provide informed consent due to cognitive or language barriers. Patients with diagnoses of schizophrenia, delusional disorder or psychotic disorder not otherwise specified (NOS), and those with traumatic brain injury, other organic brain syndromes, mental retardation, pervasive developmental disorders, dementia, or active addiction or prescription use of barbiturates, benzodiazepines, opiates, hallucinogens, stimulants, and/or cocaine at the time of appointment were also excluded from this study.

Third-year general psychiatry residents who agreed to participate (5/8) received three 50 min mentalization-based therapy (MBT) teaching sessions. Patients’ satisfaction was evaluated before and after the physician MBT training to observe if the training itself improved patient’s satisfaction scores. All patients enrolled were new to the clinic. Subsequently, patient’s satisfaction scores prior to the MBT training were acquired from a different set of patients than the patient's satisfaction scores after the MBT training. Patients were blinded to the training status of their provider.

### Diagnostic evaluations and procedures

#### Prescreening and enrollment

Psychiatry residents, in their 3rd year of residency, who consented to participate in this study attended three 50-min sessions on mentalization-based therapy (MBT) led by the same senior faculty member for all residents to assure that everyone received comparable training. Ultimately, 5 out of 8 residents chose to participate. This was a voluntary activity and demographic information from the participating residents was not specifically analyzed. The sessions consisted of discussing the basic theory of MBT, mock interviews, and homework assignments to practice mentalization with friends and family between the meetings. All new patients at the resident clinic were invited to participate in the patient satisfaction portion. Once patients were identified, study personnel reviewed their medical record and current medication list to ensure they qualified to participate. Subjects who met enrollment criteria were included in this study and were given the Patient’s Satisfaction Questionnaire (PSQ-18) after the appointment with their resident provider. Chart review was done to obtain demographic information.

#### Screening measures

*Patient’s satisfaction questionnaire (PSQ-18)* is an 18-item questionnaire designed to assess the patient’s overall satisfaction with a clinician. The PSQ-18 evaluates a patient’s perceptions of their providers technical quality, interpersonal manner, communication (doctor–patient), cost effectiveness, time spent with patient, convenience, accessibility, and overall satisfaction. The PSQ-18 was designed using 18 questions to ensure rapid completion (2–3 min) and has been used successfully in several studies with various populations to ascertain patient satisfaction [5–12].

## Results

### Patient’s description

157 (90 pre- and 67 post-physician training) patients were enrolled in this study out of an eligible pool of 209. The residents observed different patients before their training and after their training. Patients had a mean age of 41.2 (± 15.4) years. 60% of patients were married, 29% single, and 11% were either divorced or widowed. 57% of patients were employed. 33% of patients met criteria for an axis II diagnosis based on clinician assessment. There was no significant difference in demographics between the group of patients observed by residents pre- and post-mentalization training 13% versus 31% (*p* = 0.01). There was no significant difference between the patient groups in alcohol use (measured by the Alcohol Use Disorders Identification Test (AUDIT) (3.7 ± 5.2 with training vs 2.2 ± 2.5 without training; *p* = 0.088), Depressive symptom (PHQ-9) (10.9 ± 5.9 vs 11.5 ± 7.2; *p* = 0.735), or anxiety (GAD-7) (9.5 ± 5.9 vs 10.3 ± 7.2, *p* = 0.513) (see Table 1 for details).

**Table 1:** Clinical and demographic characteristics of patients evaluated by physicians with and without mentalization training

Patients	Physician’s mentalization training		*p*-value
	No (*N* = 90)	Yes (*N* = 67)	
Age	42.2 ± 15.7	39.8 ± 14.9	0.348
Gender (male)	38 (42%)	21 (31%)	0.164
Hx of abuse	28 (31%)	29 (43%)	0.117
Drug use	29 (32%)	16 (24%)	0.253
Hx of hospitalization	28 (31%)	9 (13%)	0.010
Axis II Dx	14 (16%)	19 (28%)	0.052
Substance abuse	27 (30%)	10 (15%)	0.028
AUDIT	2.2 ± 2.5	3.7 ± 5.2	0.088
PHQ-9	11.5 ± 7.2	10.9 ± 5.9	0.735
GAD-7	10.3 ± 7.2	9.5 ± 5.9	0.513

### Patient’s satisfaction questionnaire (PSQ-18)

The patients were mostly satisfied with clinician communication and interpersonal manner (PSQ-18 average scores of 4.21 ± 0.66 and 4.15 ± 0.69, respectively). To examine whether depression and anxiety ratings influenced the patients’ perception of the quality of medical care, we used Pearson correlation test for PHQ-9 and GAD-7 ratings (see Table 2). The data had a normal distribution. PSQ-18 scores on time spent (TS) and accessibility/convenience (AC) were inversely correlated with higher PHQ-9 scores (see Table 2) (*p* = 0.01 and 0.0009, respectively). This showed that if patients were more depressed, they were less likely to find that their clinicians were accessible and spent adequate time with them. Regarding anxiety symptoms, the patients score on the GAD-7 was inversely related to their scores on the accessibility and convenience (AC) subscale of the PSQ-18 (*p* = 0.01). This demonstrates that if patients were more anxious, they were less likely to find their doctor accessible.

**Table 2:** PHQ-9 and GAD-7 scores compared to PSQ-18 sub-scores

	GS	TQ	IM	Co	FA	TS	AC
PHQ-9	− 0.09774 (*p* = 0.2248)	− 0.09783 (*p* = 0.2244)	− 0.07682 (*p* = 0.3405)	− 0.11747 (*p* = 0.1441)	− 0.06006 (*p* = 0.4564)	− 0.18719 (p = 0.0193)	− 0.26408 (p = 0.0009)
GAD-7	0.03404 (*p* = 0.6908)	0.02165 (*p* = 0.8003)	0.06251 (*p* = 0.4647)	0.00669 (*p* = 0.9377)	− 0.01108 (*p* = 0.8970)	− 0.05786 (*p* = 0.4987)	− 0.20600 (p = 0.0150)

*GS* general satisfaction, *TQ* technical quality, *IM* interpersonal manner, *CO* communication, *FS* financial aspects, *TS* time spent, *AC* accessibility and convenience

**Fig. 1:**
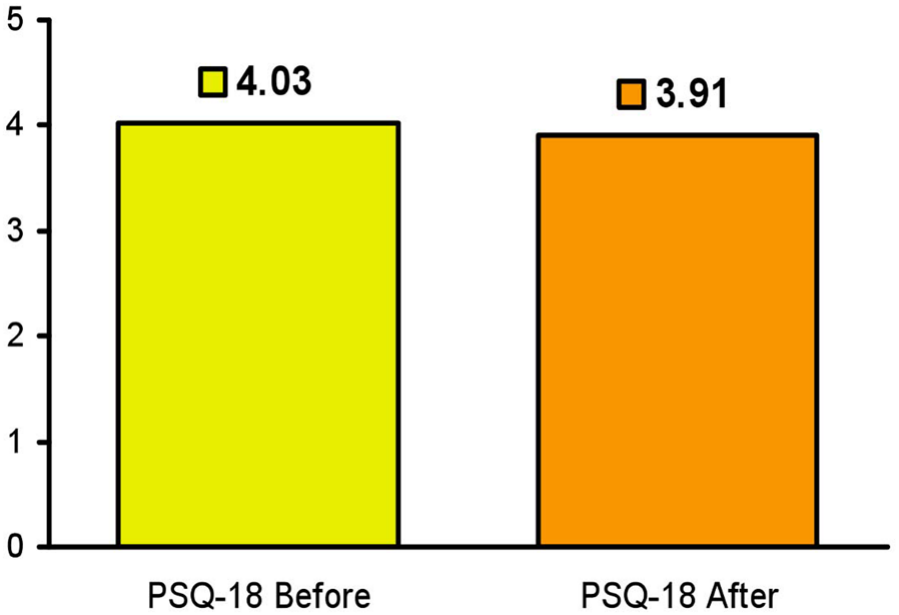
The difference between average PSQ-18 scores before and after brief resident Mentalization training. There was no difference between average patient PSQ-18 scores before and after brief resident Mentalization training (*p* = 0.439)

There was good correlation generally in all the PHQ-18 subscales as noted in Table 3. There was no significant difference between the composite PSQ-18 rating before or after the mentalization training (see Fig. 1). Generally, there was no difference in the individual subscales pre-mentalization training and post-training as noted in Table 4.

**Table 3:** PHQ-18 items

Pearson correlation coefficients, *N* = 157 Prob > |*r*| under H0: Rho = 0							
	GS	TQ	IM	Co	FA	TS	AC
GS	1.00000	0.77181 < 0.0001	0.47615 < 0.0001	0.68423 < 0.0001	0.35919 < 0.0001	0.56678 < 0.00001	0.56510 < 0.0001
TQ	0.77181 < 0.0001	1.00000	0.50125 < 0.0001	0.76321 < 0.0001	0.43091 < 0.0001	0.60912 < 0.0001	0.59782 < 0.0001
IM	0.47615 < 0.0001	0.50125 < 0.0001	1.00000	0.47306 < 0.0001	0.20053 0.0118	0.42913 < 0.0001	0.47430 < .0001
Co	0.68423 < 0.0001	0.76321 < 0.0001	0.47306 < 0.0001	1.00000	0.40703 < 0.0001	0.65662 < 0.0001	0.51900 < 0.0001
FA	0.35919 < 0.0001	0.43091 < 0.0001	0.20053 0.0118	0.40703 < 0.0001	1.00000	0.34984 < 0.0001	0.39038 < 0.0001
TS	0.56678 < 0.0001	0.60912 < 0.0001	0.42913 < 0.0001	0.65662 < 0.0001	0.34984 < 0.0001	1.00000	0.47811 < 0.0001
AC	0.56510 < 0.0001	0.59782 < 0.0001	0.47430 < 0.0001	0.51900 < 0.0001	0.39038 < 0.0001	0.47811 < 0.0001	1.00000

**Table 4:** Comparison of PSQ-18 scales by mentalization training (in the total sample, females, and males)

Measurement variable	Class variable	*n*	Mean	Median	Min	Max	sd	95% CI for mean	*p*-value
Total sample									
GS	Mentalization training = No	90	4.03	4.00	2.00	5.00	0.81	(3.86, 4.2)	0.439
	Mentalization training = Yes	67	3.91	4.00	1.00	5.00	0.88	(3.7, 4.12)	
TQ	Mentalization training = No	90	4.09	4.00	2.50	5.00	0.64	(3.96, 4.22)	0.443
	Mentalization training = Yes	67	3.98	4.00	1.75	5.00	0.68	(3.82, 4.15)	
IM	Mentalization training = No	90	4.21	4.00	2.50	5.00	0.66	(4.07, 4.34)	0.366
	Mentalization training = Yes	67	4.13	4.00	2.00	5.00	0.59	(3.98, 4.27)	
Co	Mentalization training = No	90	4.15	4.00	2.00	5.00	0.69	(4.01, 4.29)	0.086
	Mentalization training = Yes	67	3.99	4.00	1.50	5.00	0.62	(3.83, 4.14)	
FA	Mentalization training = No	90	3.65	4.00	1.00	5.00	0.98	(3.45, 3.85)	0.471
	Mentalization training = Yes	67	3.54	3.50	1.00	5.00	0.91	(3.32, 3.77)	
TS	Mentalization training = No	90	3.96	4.00	2.00	5.00	0.80	(3.79, 4.13)	0.135
	Mentalization training = Yes	67	3.77	4.00	1.50	5.00	0.76	(3.58, 3.95)	
AC	Mentalization training = No	90	3.81	3.75	2.00	5.00	0.68	(3.67, 3.96)	0.186
	Mentalization training = Yes	67	3.68	3.75	2.00	4.75	0.63	(3.53, 3.83)	

*GS* general satisfaction, *TQ* technical quality, *IM* interpersonal manner, *CO* communication, *FS* financial aspects, *TS* time spent, *AC* accessibility and convenience

## Discussion

Our study’s goal was to evaluate patient satisfaction in an outpatient psychiatry resident clinic and support the premise that mentalization training can be a helpful part of improving patient satisfaction. Our study shows that the patients observed in the general outpatient clinic had an overall favorable impression of their clinicians even prior to MBT training (average was a 4/5). However, our study also shows that patients observed in an outpatient psychiatry clinic are more likely to be displeased with time-related parameters. Consistent with previously cited studies, depressed patients are more likely to be impatient and frustrated with both the accessibility and the amount of time their clinician spends with them. Furthermore, anxious patients are more likely to find their physicians inaccessible [6–15, 21, 22]. Given these results, the use of patient satisfaction questionnaires to measure physician performance in a setting where anxiety and depression dominate the patients presenting symptomology should be administered and interpreted with extreme caution (especially in accessibility and time spent parameters).

This study was intended to be a brief introduction to mentalization techniques and not a comprehensive tutelage on the complex subject. The intervention was designed to be simple and short to accommodate practical didactic limitations of an average residency training program. Although our brief training of residents in mentalization strategies did not significantly improve their already high patient rating scale results, we were able to show that mentalization strategies can be easily incorporated into the residency didactic setting.

## Limitations

Regarding the correlation between depression, anxiety, and patient satisfaction, this study was limited by the relatively small sample size, limited single assessment of patient satisfaction, and lack of information on other factors that could be limiting the level of satisfaction (i.e., pain). Regarding the assessment of the impact of mentalization training, this study had significant limitations. The relatively short intervention of three educational sessions might have contributed to the lack of PSQ-18 improvement post-training. It is reasonable to hypothesize that the non-significant trends in PSQ-18 scores noted would have reached significance had the resident training been more extensive. It could also be argued that the PSQ-18 was not the best way to evaluate the physician’s ability to build a good therapeutic alliance through mentalization strategies. Another limitation was that we did not measure the resident’s degree of mastery of the mentalization strategy. It is possible that despite being taught the techniques, they did not learn them well enough to put them into practice. Another important source of bias to consider is that mentalization training was based on resident interest (vs. randomizing trainees to training or no training).

## Conclusions

Despite these limitations, this study supports the findings of previous studies showing that individuals with depression and anxiety are more likely to be less satisfied with their providers and highlights the difficulties of measuring patient satisfaction in patients with depression and anxiety. This study was unable to demonstrate improvement in patient satisfaction after providers received brief mentalization training. However, this study introduces an approach to defining and measuring the benefits of mentalization training for psychiatry residents that was effectively implemented into the resident’s curriculum.

### Suggestions for future studies

Future studies should try and asses more robust mentalization training to a larger, more diverse physician population. Furthermore, future studies should add an evaluation of learner’s mastery of the concepts to observe if it correlates to improvements in outcome measure. Other studies might also include other outcome measures besides the PSQ-18 to evaluate the quality of the doctor–patient interaction.

## Data Availability

The datasets during and/or analyzed during the current study are available from the corresponding author on reasonable request.

## Abbreviations

ACA: Affordable Care Act
AC: Accessibility and convenience
AUDIT: Alcohol Use Disorders Identification Test
CO: Communication
GAD-7: Generalized Anxiety Disorder 7-Item scale
FS: Financial aspects
GS: General satisfaction
IRB: Internal review board
IM: Interpersonal manner
MBT: Mentalization-based therapy
NOS: Not otherwise specified
PSQ-18: Patient Satisfaction Questionnaire
PHQ-9: Patient Health Questionnaire
TQ: Technical quality
TS: Time spent
